# Hydrogen Sulfide Primes bZIP68 via Persulfidation to Enhance Redox-Dependent Transcription and Adaptation to Osmotic Stress in Rice

**DOI:** 10.3390/ijms27093841

**Published:** 2026-04-26

**Authors:** Xiaoyun Ma, Fengchao Zhai, Lingxi Geng, Guojing Chen, Wenge Li, Mohammad Saidur Rhaman, Jing Zhang, Yanjie Xie, Heng Zhou

**Affiliations:** 1 Laboratory Center of Life Sciences, College of Life Sciences, Nanjing Agricultural University, Nanjing 210095, China; 2022216007@stu.njau.edu.cn (X.M.); 2021216043@stu.njau.edu.cn (F.Z.); 9201010221@stu.njau.edu.cn (L.G.); 2023116080@stu.njau.edu.cn (G.C.); 2025116081@stu.njau.edu.cn (W.L.); 2 Department of Seed Science and Technology, Bangladesh Agricultural University, Mymensingh 2202, Bangladesh; saidursst@bau.edu.bd; 3 National Key Laboratory for the Development and Utilization of Forest Food Resources, Co-Innovation Center for Sustainable Forestry in Southern China, State Key Laboratory of Tree Genetics and Breeding, Key Laboratory of State Forestry and Grassland Administration on Subtropical Forest Biodiversity Conservation, College of Life Sciences, Nanjing Forestry University, Nanjing 210037, China; jingzhang@njfu.edu.cn

**Keywords:** persulfidation, bZIP68, rice, osmotic stress, transcriptional reprogramming

## Abstract

Osmotic stress limits rice productivity, yet the crosstalk between hydrogen sulfide signaling and redox regulation remains incompletely understood. We previously showed that redox-dependent oligomerization of the basic (region) leucine zippers transcription factor bZIP68 at Cys245 confers osmotic tolerance. However, the role of an adjacent cysteine, Cys171, was undefined. Here, we demonstrate that osmotic stress induces persulfidation of bZIP68 specifically at Cys171. This modification facilitates Cys245-mediated oxidation-dependent oligomerization, thereby enhancing bZIP68 transcriptional activity toward *COLD-REGULATED413-THYLAKOID MEMBRANE1* (*COR413-TM1*). Transgenic complementation and physiological assays confirmed that Cys171 persulfidation is essential for full bZIP68 function in osmotic adaptation. Transcriptomic analysis further revealed that Cys171 is required for bZIP68-driven transcriptional reprogramming under stress. Our findings establish a hierarchical redox cascade wherein persulfidation primes bZIP68 for oxidative activation, highlighting a regulatory crosstalk between distinct post-translational modifications. These mechanistic insights expand our understanding of H_2_S signaling and identify the bZIP68 cysteine network as a potential target for improving crop stress resilience.

## 1. Introduction

Plants are constantly exposed to fluctuating environmental conditions that impose profound constraints on growth and productivity. Among these, osmotic stresses such as drought and salinity are particularly pervasive, disrupting cellular homeostasis and rapidly triggering excessive accumulation of reactive oxygen species (ROS) [1]. Although ROS uncontrolled accumulation leads to oxidative damage to proteins, lipids, and nucleic acids, they also act as important signaling molecules [2]. Consequently, effective stress adaptation requires not only activation of antioxidant systems but also precise reprogramming of redox-sensitive signaling networks that coordinate gene expression with cellular metabolic and redox states.

Transcription factors (TF) represent key nodes for integrating redox signals into stress-responsive gene expression. Several plant transcription factors, including members of the bZIP, NAC, and MYB families, are known to be regulated by oxidative post-translational modifications (oxiPTMs), such as disulfide bond formation, S-nitrosylation, or oxidation of specific cysteine residues [3,4,5,6]. These redox modifications often control protein oligomerization and DNA-binding affinity, thereby enabling rapid and reversible tuning of transcriptional outputs in response to changing environmental conditions [7]. Previous studies have shown that bZIP68 is a stress-responsive basic leucine zipper transcription factor in rice that plays a critical role in osmotic stress tolerance by regulating the expression of genes involved in redox homeostasis and stress defense [8]. Under osmotic stress conditions, the accumulation of ROS was perceived by redox sensor thiol peroxidase (GPX1) and then transduced into bZIP68. bZIP68 was quickly oxidized by GPX1, and this led to the oligomerization and transcriptional activation of bZIP68. However, whether persulfidation participates in such redox control of bZIP68, and how it mechanistically interfaces with redox signaling, remain largely unexplored.

Hydrogen sulfide (H_2_S) has emerged as a key gaseous signaling molecule involved in diverse physiological processes, including seed germination, root development, stomatal movement, and responses to abiotic stresses [9,10,11,12,13]. In plant stress biology, H_2_S is generally associated with enhanced stress tolerance and redox protection, often through modulation of antioxidant capacity [14]. At the molecular level, a central mechanism of H_2_S signaling is protein persulfidation, a reversible post-translational modification that converts cysteine thiols (-SH) into persulfides (-SSH), thereby altering protein activity, stability, or interactions [15,16,17]. Large-scale persulfidomic studies have identified hundreds of persulfidated proteins in plants, implicating H_2_S in broad regulation of metabolism, hormone signaling, and stress responses [18,19,20,21,22,23]. Recent studies have reported the involvement of H_2_S-mediated persulfidation in the ABA signaling pathway [24,25,26]. Despite these advances, the current understanding of H_2_S signaling remains largely phenomenological. Most studies describe H_2_S as a general activator of antioxidant defenses or a global redox modulator [12], while the mechanistic principles by which H_2_S selectively rewires signaling pathways remain poorly defined. In particular, how persulfidation is integrated with classical redox signaling networks is still an open question.

In this study, we aim to elucidate the function of H_2_S-mediated persulfidation in controlling stress adaptation of rice plants. We found that the redox-sensitive TF, bZIP68, undergoes persulfidation both in vivo and in vitro. Further biochemical evidence revealed that Cys171 is a key persulfidation site on bZIP68, and this modification facilitates subsequent oxidation-dependent oligomerization of bZIP68, thereby enhancing its transcriptional activity in response to osmotic stress. By combining genetic and transcriptomic analyses, we provide evidence here that persulfidation of bZIP68 amplifies stress-induced gene expression and confers enhanced tolerance to osmotic stress in rice. Together, our findings provide a mechanistic framework for understanding how different oxiPTMs cooperated in fine-tuning transcription reprogramming during plant stress responses and a genetic resource for breeding stress-resistant rice varieties.

## 2. Results

### 2.1. bZIP68 Undergoes Persulfidation Both In Vitro and In Vivo

**Figure 1:**
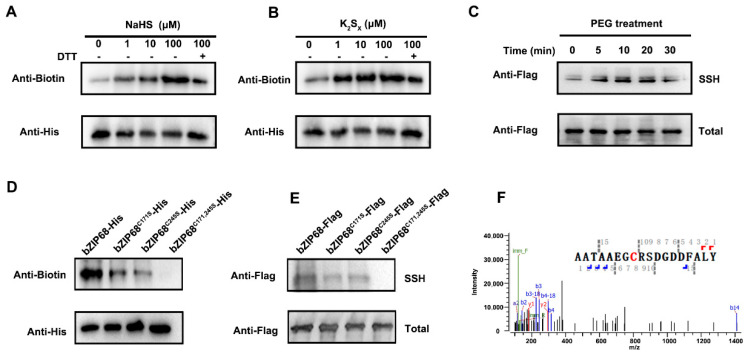
bZIP68 is regulated by persulfidation. (**A**) In vitro, bZIP68-His recombinant protein was treated with NaHS (0–100 μM) for 30 min to induce persulfidation, with 10 mM DTT treatment as a negative control. After tag-switch labeling, persulfidated proteins (SSH) were detected using an anti-biotin antibody, and total bZIP68-His protein was detected using an anti-His antibody as a loading control. (**B**) In vitro, bZIP68-His recombinant protein was treated with K_2_S_x_ (0–100 μM) for 30 min to induce persulfidation. After tag-switch labeling, SSH was detected using an anti-biotin antibody, and total bZIP68-His protein was detected using an anti-His antibody as a loading control. (**C**) In vivo, the bZIP68-Flag fusion protein in rice protoplasts was induced to undergo persulfidation by PEG treatment for different durations (0–30 min). After tag-switch labeling and streptavidin magnetic bead purification, SSH was detected using an anti-Flag antibody, and total bZIP68-Flag protein without streptavidin magnetic bead purification was used as a loading control. (**D**) In vitro, persulfidation analysis of wild-type (WT) bZIP68-His and its cysteine mutants (C171S, C245S, C171/245S) induced by NaHS was performed. After tag-switch labeling, SSH was detected using an anti-biotin antibody, and total protein was detected using an anti-His antibody as a loading control. (**E**) In vivo, persulfidation analysis of WT bZIP68-Flag and its cysteine mutants (C171S, C245S, C171/245S) in rice protoplasts induced by PEG treatment was performed. After tag-switch labeling and streptavidin magnetic bead purification, SSH was detected using an anti-Flag antibody, and total bZIP68-Flag protein was used as a loading control. (**F**) A fully annotated MS/MS spectrum of a bZIP68 peptide containing a persulfidated cysteine confirmed that Cys171 is the persulfidation site of bZIP68.

Our previous study has shown that bZIP68 is a redox-sensitive TF and is easily oxidized in response to osmotic stress [8]. Given the involvement of H_2_S in rice osmotic stress responses, we hypothesized that bZIP68 might also be regulated by persulfidation. To test this, we first examined persulfidation of bZIP68 in vitro using purified recombinant bZIP68-His protein. Treatment with increasing concentrations of NaHS or K_2_S_x_ led to a clear, dose-dependent increase in bZIP68 persulfidation signals, whereas DTT treatment significantly decreased the persulfidation level of bZIP68 (Figure 1A,B), indicating that bZIP68 can be directly persulfidated by H_2_S donors.

To examine whether bZIP68 undergoes persulfidation in vivo, we transiently transfected rice protoplasts with a *bZIP68-Flag* construct and performed Tag-switch analysis with anti-Flag antibody. Notably, a rapid increase in bZIP68 persulfidation within 10 min after PEG treatment was observed (Figure 1C). Since our previous study showed that drought treatment could significantly increase the endogenous H_2_S accumulation in rice plants [27], these results suggested that osmotic stress triggers persulfidation of bZIP68 in planta.

bZIP68 contains two cysteine residues, Cys171 and Cys245. To identify the persulfidation cysteine residues, we mutated them to serine (Ser) individually or together. In both in vitro and in vivo assays, mutation of either Cys171 or Cys245 partially reduced persulfidation, while the double mutant (C171, 245S) almost completely abolished the persulfidation signal (Figure 1D,E), demonstrating that both Cys171 and Cys245 are modified. Furthermore, LC-MS/MS analysis confirmed that Cys171 is a bona fide persulfidation site (Figure 1F). Together, these results demonstrate that bZIP68 undergoes dynamic persulfidation at Cys171 and Cys245 in response to osmotic stress.

### 2.2. Persulfidation at Cys171 Facilitates Oxidative Oligomerization of bZIP68

**Figure 2:**
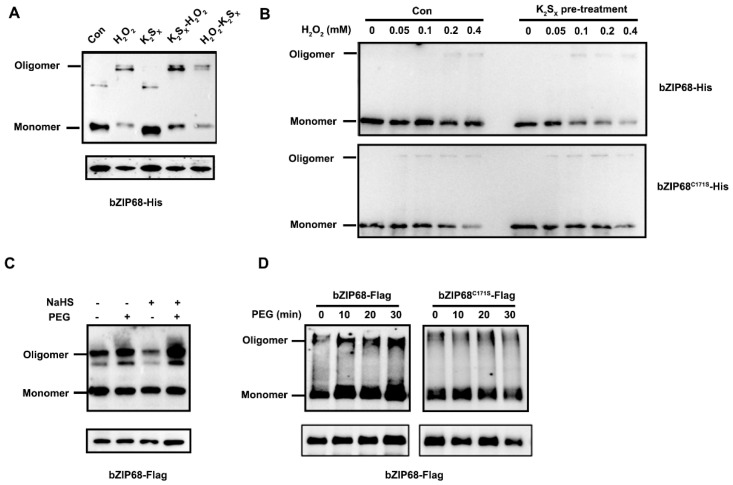
Persulfidation of Cys171 facilitates oligomerization of bZIP68. (**A**) Western blotting analysis of the oligomerization state of bZIP68-His recombinant protein under different treatments (Con, K_2_S_x_, K_2_S_x_ + H_2_O_2_, H_2_O_2_). bZIP68-His was treated with 0.2 mM H_2_O_2_ or 0.1 mM K_2_S_x_ alone for 30 min, or after that, treated with or without 0.2 mM H_2_O_2_ or 0.1 mM K_2_S_x_ alone for 30 min again and then the protein was harvested for immunoblot analysis. The lower bands indicate total monomer bZIP68-His protein, serving as a loading control. (**B**) The effects of different concentrations of H_2_O_2_ (0, 0.05, 0.1, 0.2, 0.4 mM) treatment for 5 min on the oligomerization of bZIP68-His and bZIP68^C171S^-His recombinant proteins, and the changes after 0.1 mM K_2_S_x_ pretreatment for 30 min. (**C**) The effects of NaHS and PEG treatment on the oligomerization state of bZIP68-Flag fusion protein in rice protoplasts. Rice protoplasts expressing bZIP68-Flag were treated with or without 0.1 mM NaHS for 30 min, and then treated with or without 20% PEG 6000 for 20 min, and then the protein was harvested for immunoblot analysis. The lower bands indicate total monomer bZIP68-Flag protein, serving as a loading control. (**D**) The dynamic effects of PEG treatment over different times (0, 10, 20, 30 min) on the oligomerization of bZIP68-Flag and bZIP68^C171S^-Flag fusion proteins in rice protoplasts. The lower bands show total monomer bZIP68-Flag protein, serving as a loading control.

Since bZIP68 could form oligomers through Cys245-dependent oxidation [8], we therefore asked whether persulfidation influences this oxidation-driven oligomerization process. Accordingly, bZIP68-His proteins were pretreated with or without K_2_S_x_, and then treated with H_2_O_2_. Immunoblot analysis revealed that H_2_O_2_ alone induced moderate oligomerization of bZIP68, whereas K_2_S_x_ pretreatment markedly enhanced H_2_O_2_-induced oligomer formation (Figure 2A). Importantly, K_2_S_x_ treatment alone had no effects on the oxidation status of bZIP68, indicating that persulfidation itself does not directly cause oligomer formation, but instead sensitizes bZIP68 to subsequent oxidation. Consistently, dose–response experiments showed that bZIP68 oligomerization occurred at 0.2 mM H_2_O_2_ for 5 min treatment, whereas after K_2_S_x_ pretreatment, oligomerization was already detectable at 0.1 mM H_2_O_2_ (Figure 2B). In contrast, the bZIP68^C171S^ mutant displayed oligomerization at much lower H_2_O_2_ concentrations even without K_2_S_x_ pretreatment, and K_2_S_x_ no longer affected its oxidation sensitivity (Figure 2B), indicating that Cys171 is the critical residue mediating persulfidation-enhanced bZIP68 oxidation.

To determine whether this phenomenon could happen in vivo, the rice protoplasts transiently expressing *bZIP68-Flag* or *bZIP68^C171S^-Flag* construct were pretreated with or without NaHS and then subject to PEG treatment. Immunoblot analysis revealed that NaHS pretreatment significantly increased PEG-induced oligomerization of bZIP68 in rice protoplasts (Figure 2C). Moreover, PEG treatment led to a progressive accumulation of bZIP68 oligomers, whereas bZIP68^C171S^ showed no significant change in oligomerization dynamics (Figure 2D). These results demonstrate that persulfidation at Cys171 promotes osmotic stress-induced oligomerization of bZIP68.

### 2.3. Persulfidation at Cys171 Enhances the Transactivation Activity of bZIP68

**Figure 3:**
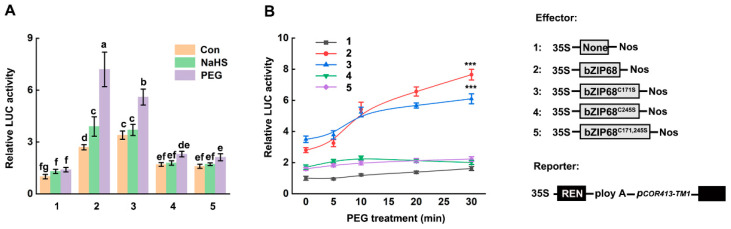
Persulfidation of Cys171 enhances the transactivation activity of bZIP68. (**A**) Dual-luciferase reporter assay detecting the effect of different treatments (100 μM NaHS, 20%PEG6000 for 30 min) on the transcriptional activation activity of the *COR413-TM1* gene promoter by wild-type *bZIP68* and its cysteine mutants. Data represent the mean ± standard deviation of three independent experiments. Different letters above the bars indicate significant differences between groups (*p* < 0.05, one-way ANOVA followed by Tukey’s multiple comparison test). (**B**) Dual-luciferase reporter assay detecting the dynamic changes in target gene transcriptional activation by wild-type *bZIP68* and its cysteine mutants under PEG treatment at different time points (0–30 min). Data represent the mean ± standard deviation of three independent experiments. *** indicates an extremely significant difference compared with the empty vector control (*p* < 0.001).

To determine whether persulfidation affects bZIP68 transcriptional activity, we performed dual-luciferase reporter assays using the promoter of *COLD-REGULATED413-THYLAKOID MEMBRANE1* (*COR413-TM1*) as a target [8]. Compared with the control condition, PEG treatment significantly enhanced the transcriptional activation activity of bZIP68 and bZIP68^C171S^, but not bZIP68^C245S^ or bZIP68^C171,245S^, indicating the importance of Cys245 in bZIP68 function. Interestingly, PEG treatment-induced enhancement of the transcriptional activation activity in bZIP68 was significantly attenuated in bZIP68^C171S^, indicating that Cys171 was also responsible for the activation of bZIP68 in response to osmotic stress. Moreover, NaHS treatment significantly enhanced the transcriptional activation activity of wild-type bZIP68, but had no effect on bZIP68^C171S^, bZIP68^C245S^ or the bZIP68^C171,245S^ (Figure 3A), indicating persulfidation of Cys171 enhances the transactivation activity of bZIP68. Together, these results indicated that persulfidation of Cys171 is responsible for the full activation of bZIP68 under osmotic stress.

Further time-course analyses revealed that wild-type bZIP68 showed a gradual increase in transactivation activity under PEG treatment, peaking around 30 min, whereas bZIP68^C171S^ displayed higher activity than bZIP68 before 10 min but lower activity after 10 min treatment (Figure 3B). Combined with their oligomerization status (Figure 2), these results suggested that Cys171 persulfidation is required for the oligomerization of bZIP68 and the enhancement of its transcriptional activity under osmotic stress.

### 2.4. Persulfidation-Deficient bZIP68 Mutants Compromise Osmotic Stress Tolerance in Rice

**Figure 4:**
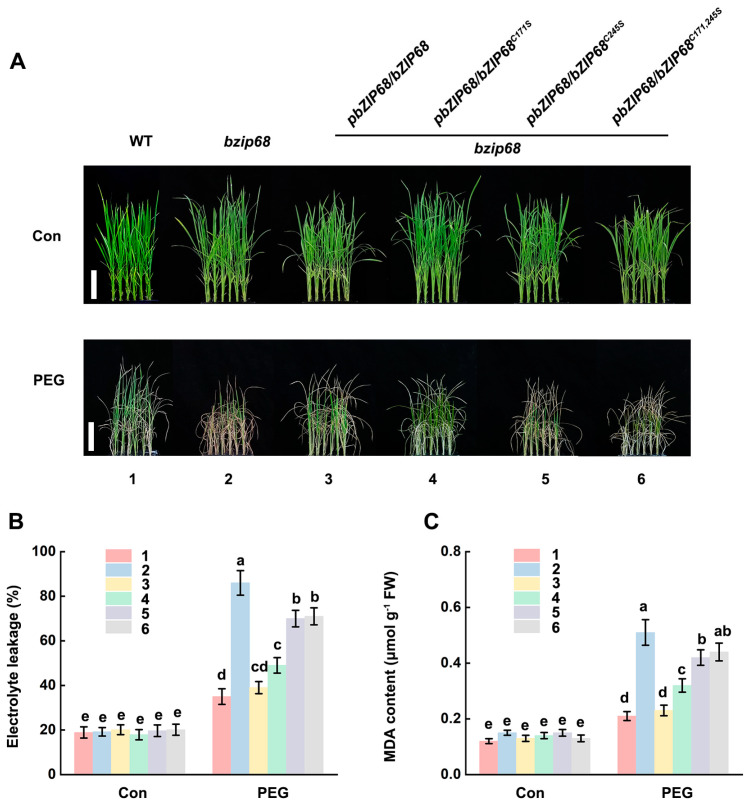
Cys171 of bZIP68 mediates rice plant responses to osmotic stress. Phenotypic analysis of osmotic stress tolerance in wild-type plants, bzip68 mutants, bZIP68 full-length complementation lines and bZIP68 point-mutated complementation lines. Two-week-old seedlings of wild-type plants, *bzip68* mutants, *pbZIP68*/*bzip68* complementation lines, *pbZIP68^C171S^*/*bzip68*, *pbZIP68^C245S^*/*bzip68* and *pbZIP68^C171^*^/*245S*^/*bzip68* point-mutated complementation lines were treated with 20% PEG6000 for 6 days, after which the growth phenotypes were observed (**A**), and electrolyte leakage (**B**) and malondialdehyde (MDA) content (**C**) were determined. Experiments were repeated at least three times with similar results. Data are presented as mean ± SD (n = 3). Different letters denote significant differences at *p* < 0.05 (one-way ANOVA, Duncan’s multiple range test).

To evaluate the biological significance of bZIP68 persulfidation, we generated complementation lines expressing wild-type or cysteine-mutated *bZIP68* under the control of its native promoter in the *bzip68* mutant background (*pbZIP68*/*bzip68*, *pbZIP68^C171S^*/*bzip68*, *pbZIP68^C245S^*/*bzip68*, and *pbZIP68^C171^*^,*245S*^/*bzip68*). By using RT-qPCR analysis, the transgenic plants with the *bzip68* background exhibited a similar expression level of bZIP68, which was used for further experiments (Figure S1). Under normal conditions, all lines showed comparable growth. Under 20% PEG treatment, *bzip68* mutants exhibited severe growth inhibition compared to wild-type plants (Figure 4A). Complementation with wild-type *bZIP68* fully rescued the stress-sensitive phenotype of the *bzip68* mutant, whereas *bZIP68^C171S^* only partially rescued the *bzip68* mutant phenotype observed upon PEG treatment. By contrast, the introduction of either *bZIP68^C245S^* or the *bZIP68^C171^*^,*245S*^ barely rescued the *bzip68* mutant phenotype (Figure 4A). Consistently, PEG-treated *bzip68* mutants showed significantly higher electrolyte leakage and MDA accumulation, indicating enhanced membrane damage and lipid peroxidation (Figure 4B,C). These physiological defects were fully corrected by wild-type *bZIP68*, partially rescued by *bZIP68^C171S^*, barely rescued by *bZIP68^C245S^* or the *bZIP68^C171^*^,*245S*^. These results demonstrate that Cys171 is also required for the full protective function of bZIP68 during osmotic stress.

### 2.5. Persulfidation of Cys171 Is Required for bZIP68-Mediated Transcriptional Reprogramming

To identify potential direct transcriptional targets of bZIP68 in response to osmotic stress, we conducted comparative transcriptome analysis via RNA sequencing (RNA-seq) on two-week-old *bzip68* mutant, *pbZIP68/bzip68*, and *pbZIP68^C171S^/bzip68* seedlings treated with or without 20% PEG 6000 for 12 h. We then identified differentially expressed genes (DEGs) based on the criteria of a significant difference (*p* < 0.05) with an absolute fold-change ≥ 2.

**Figure 5:**
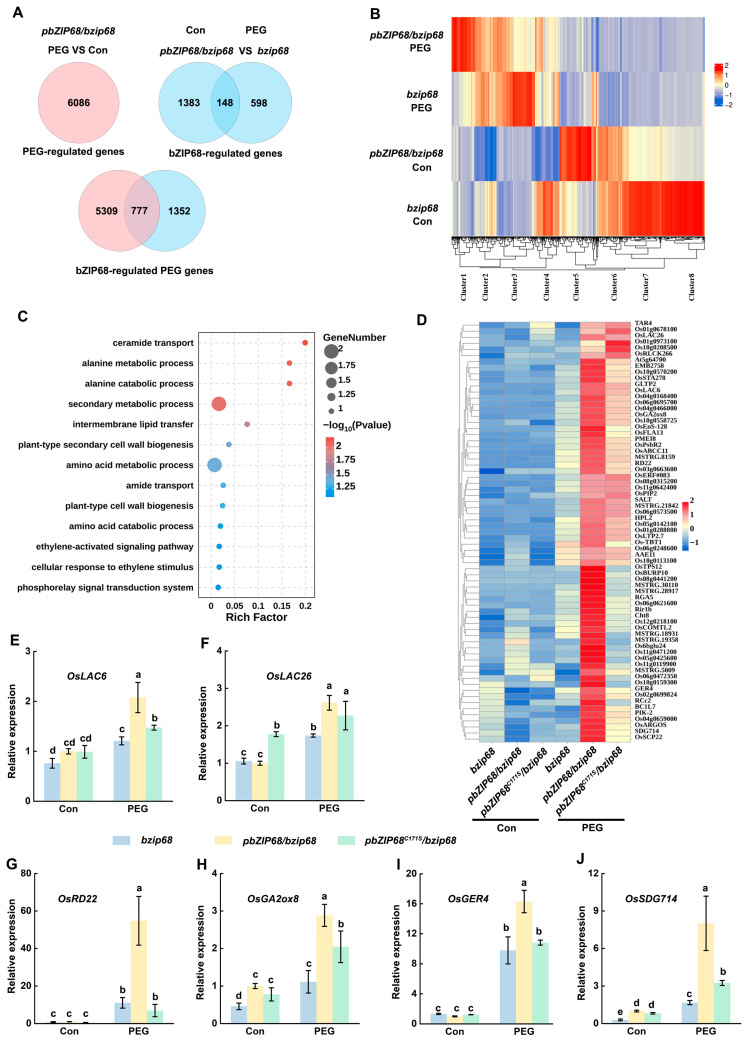
Transcriptional analysis of bZIP68-dependent stress-regulated genes. (**A**) Venn diagrams representing DEGs regulated by PEG (top, left), bZIP68 (top, right), and both PEG and bZIP68 (bottom). (**B**) Clustering analysis of bZIP68-regulated stress-responsive genes. The heatmap shows the Log2 (TPM) values of 777 DEGs. DEGs with similar expression patterns were clustered with the R package Pheatmap. (**C**) GO analysis showing the most enriched terms of Clusters 1, shown in B. Bubble charts show the GO terms. (**D**) Heatmap showing the Log2 (TPM) of enriched genes in Cluster 1. (**E**–**J**) Relative gene expression in bzip68, *pbZIP68*/*bZIP68*, and *pbZIP68^C171S^*/*bZIP68* seedlings before and after PEG treatment, as determined by RT-qPCR. Expression in the untreated *pbZIP68*/*bZIP68* was set to 1.00. Each bar represents the mean of three independent experiments ± SD. Different letters represent significant differences (*p* < 0.05, one-way ANOVA).

A total of 6086 DEGs were identified as PEG-regulated DEGs in *pbZIP68*/*bzip68* seedlings after exposure to PEG treatment compared with seedlings before treatment, among which 3296 were upregulated, and 2790 were downregulated (Figure 5A; Table S1). To identify bZIP68-regulated DEGs, we compared the RNA-seq results from the bzip68 mutant and *pbZIP68*/*bzip68* with or without PEG treatment, leading to the identification of 2129 DEGs regulated by bZIP68, including 1531 DEGs at the control condition,746 DEGs at PEG treatment (Tables S2 and S3). A comparison of 6086 and 2129 DEGs defined a total of 777 bZIP68-regulated osmotic stress-responsive genes (Figure 5A; Table S4).

Gene ontology (GO) analysis revealed that these genes were significantly enriched in biological processes, including plant-type secondary cell wall biogenesis, response to oxidative stress, and regulation of the cell cycle process, supporting a key role of bZIP68 in rice osmotic stress adaptation (Table S5). Moreover, cluster analysis grouped these 777 bZIP68-regulated osmotic stress-responsive genes into eight clusters (Clusters 1–8) (Figure 5B). Among these, the DEGs in cluster 1 exhibited a higher expression level in *pbZIP68*/*bzip68* under PEG treatment than in the *bzip68* mutant (Table S6). GO analysis showed that cluster 1 is enriched in biological processes involved in secondary metabolic processes, plant-type cell wall biogenesis, and cellular response to ethylene stimulus (Figure 5C), indicating that bZIP68 may confer rice osmotic stress tolerance by regulating cell wall biogenesis and hormone signaling.

To explore the function of Cys171 on bZIP68-regulated transcriptional reprogramming, we analyzed DEGs grouped in cluster 1 by constructing heatmaps. Notably, the heatmaps showed that the expression of several stress responsive genes including OsLAC6 (Laccase 6) [28], OsLAC26 (Laccase 26) [29], OsGA2ox8 (Gibberellin 2-oxidase 8) [30], OsRD22 (Dehydration-responsive gene 22) [31], OsGER4 (Germin-like protein 4) [32], and OsSDG714 (for SET Domain Group Protein714, encodes a histone H3K9-specific methyltransferase) [33] were highly induced by PEG treatment in *pbZIP68*/*bzip68*, while slightly induced in *pbZIP68^C171S^*/*bzip68* (Figure 5D), indicating that Cys171 is important for the bZIP68 transcription activity.

To verify the transcriptome data, we further performed the qRT-PCR analysis. The results confirmed that all six genes were significantly upregulated by PEG treatment in *pbZIP68*/*bzip68* seedlings, whereas their induction was markedly attenuated in the *pbZIP68^C171S^*/*bzip68* (Figure 5E–J). These findings further indicate that persulfidation of Cys171 is required for bZIP68-mediated transcriptional reprogramming in rice response to osmotic stress.

## 3. Discussion

In response to various stresses, plants have evolved a variety of complex defense systems of signal perception and transduction networks [34,35]. Transcription factors (TFs) that are activated by different pathways of signal transduction and can directly or indirectly combine with cis-acting elements to modulate the transcription efficiency of target genes, which play key roles in crop genetic improvement [36]. Over the past decade, significant progress has been made in deciphering the role of plant TFs as key regulators of environmental responses, particularly in important cereal crops [37]. However, how the activity or function of TFs was regulated to fine-tune the transcription reprogramming in response to changing environmental conditions remains unclear. In this study, we reported a synergistic regulatory mechanism composed of persulfidation and oxidation in the activation of bZIP68 function in rice osmotic stress response. Under osmotic stress, substantial H_2_S accumulation leads to persulfidation of bZIP68 at both Cys171 and Cys245, a redox-sensitive TF that acts in osmotic stress response. Furthermore, the persulfidation of Cys171 is required for osmotic stress-induced oligomerization and enhancement of transcriptional activity of bZIP68. The identification of bZIP68 as a direct persulfidation target function in the facilitation of stress-induced oligomerization of bZIP68 in response to osmotic stress contributes to our understanding of the role of protein persulfidation in stress-mediated transcription reprogramming. Furthermore, this study strongly suggests that bZIP68 plays a pivotal role as the convergence point for both H_2_S and ROS signaling, thereby fine-tuning plant stress responses.

Oxidative post-translational modifications (PTMs), including S-sulfenylation, S-nitrosation, and persulfidation, have been reported to affect the activity of TFs in plants, providing a robust and flexible mechanism for metabolic, genetic, and epigenetic regulation in response to environmental cues [7]. OxiPTMs can influence distinct aspects of TF function, such as their subcellular localization, stability/structure, protein–protein interactions, and DNA binding capacity, thereby resulting in gene expression reprogramming. Our previous study showed that bZIP68, a VRE-like bZIP TF, positively regulates rice osmotic response by binding and activating a series of osmotic-responsive genes. Moreover, bZIP68 is easily oxidized, and the Cys171 and Cys245 of bZIP68 undergo sulfenylation (-SOH) under osmotic stress conditions. Considering H_2_S can react with oxidized thiol derivatives (e.g., disulfides or -SOH) to form persulfides (-SSHs) [12], we wonder whether bZIP68 can be persulfidated in response to osmotic stress. In this study, several lines of evidence indicate that the persulfidation of bZIP68 occurs in response to osmotic stress. In vitro tag-switch assays, together with site-directed mutagenesis of the cysteine residues, illustrated that bZIP68 undergoes persulfidation at Cys171 and Cys245 (Figure 1). This was further corroborated by an in vivo tag-switch assay, which showed that the persulfidation level of bZIP68 was decreased when Cys171 or Cys245 was mutated. These results further indicated that bZIP68 is a redox-sensitive protein. Moreover, the increased persulfidation level under PEG treatment was observed, indicating that persulfidation may be involved in the function regulation of bZIP68 in response to osmotic stress.

Oxidation promotes the homo-tetramerization of bZIP68, of which Cys245 is the key residue. Notably, homo-tetramerization is required for the increase in DNA binding capability and transcriptional activity of bZIP68 in vivo. To investigate the consequence of persulfidation on bZIP68 function, we first check whether persulfidation affects the redox status of bZIP68. Interestingly, pretreatment of K_2_S_X_ not only facilitates the H_2_O_2_-induced oligomerization of bZIP68, but also enhances its oxidation sensitivity, as dose–response experiments showed that bZIP68 oligomerization occurred at 0.2 mM H_2_O_2_ for 5 min treatment, whereas after K_2_S_x_ pretreatment, oligomerization was already detectable at 0.1 mM H_2_O_2_ (Figure 2B). These results indicated that persulfidation may affect the bZIP68 structure and then enhance its affinity to H_2_O_2_. Sulfides can react with disulfides to form persulfides, thus may dissociate a homodimer or oligomer formed by an intermolecular disulfide bond [15]. Notably, K_2_S_X_ has no effects on the oligomer of bZIP68. It may be due to the unsuitable space structure of the Cys245-based disulfide bond in the bZIP68 oligomer for sulfide reaction. Further in vivo evidence showed that NaHS pretreatment also significantly increased PEG-induced oligomerization of bZIP68 (Figure 2C). Moreover, PEG treatment led to a progressive accumulation of bZIP68 oligomers, whereas bZIP68^C171S^ showed no significant change in oligomerization dynamics under PEG treatment (Figure 2D). These results demonstrate that persulfidation at Cys171 is responsible for osmotic stress-induced oligomerization of bZIP68. Such a priming mechanism may represent a general principle of H_2_S signaling. Many proteomic data have shown that redox-sensitive proteins often undergo multiple oxiPTMs, including persulfidation, S-nitrosylation and sulfenylation [38,39]. The coexistence of multiple cysteine-based modifications suggests that in some cases, persulfidation may function as a “molecular preconditioning” step that lowers the activation threshold for subsequent oxidative regulation. In this context, H_2_S would act not as a dominant signal but as a fine-tuner that calibrates the dynamic range and sensitivity of redox-responsive proteins.

The oligomerization is an important regulatory mechanism for bZIP68-mediated enhancement of transcriptional activity in response to osmotic stress. We found that PEG treatment-induced enhancement of the transcriptional activation activity in bZIP68 was significantly attenuated in bZIP68^C171S^. indicating the importance of Cys171 in bZIP68 activity. We also observed NaHS treatment significantly enhanced the transcriptional activation activity of wild-type bZIP68, but had no effect on bZIP68^C171S^, bZIP68^C245S^ or the bZIP68^C171,245S^ (Figure 3A), indicating persulfidation of Cys171 enhances the transactivation activity of bZIP68. As osmotic stress rapidly increased the persulfidation level of bZIP68, these results indicated that persulfidation of Cys171 is responsible for the full function of bZIP68 under osmotic stress. Further transgenic validation and measurements of multiple physiological traits demonstrated persulfidation of Cys171 is required for the bZIP68-mediated osmotic stress tolerance (Figure 4). Transcriptomic analyses also revealed that persulfidation of Cys171 is required for bZIP68-mediated transcriptional reprogramming in rice response to osmotic stress. Together, these results establish persulfidation as an essential regulatory modification that enables bZIP68 to efficiently mediate stress-adaptive transcriptional responses.

Previous study also reported that H_2_S-mediated persulfidation of ABI4 enhances its transactivation activity toward *Mitogen-Activated Protein Kinase Kinase 18* in the regulation of plant responses to ABA [26]. Moreover, persulfidation of ABI4 alleviates its degradation triggered by the 26S proteasome pathway during seed germination [40]. Thus, it is worth determining whether persulfidation plays a role in regulating bZIP68 protein stability.

The regulation of transcription factors by redox modifications is emerging as a central theme in plant stress biology. Several transcription factors, such as BZR1, GmNTL1, and various bZIP members, are known to undergo redox-dependent conformational changes that control DNA binding or transcriptional activity [3,4,5,8]. Our findings extend this paradigm by introducing persulfidation as an additional layer of regulation that precedes and facilitates oxidation-induced activation. This layered regulatory architecture may provide plants with a flexible and robust mechanism to integrate multiple environmental and metabolic signals into coherent transcriptional responses.

## 4. Materials and Methods

### 4.1. Plant Materials and Growth Conditions

The Rice (*Oryza sativa* L.) used in this study was the japonica cultivar Wuyungeng 7 or derived from this cultivar. For complementation lines of *bZIP68* and its mutant variants, the *1300221-Flag* vector was used as the backbone. A 2000 bp promoter sequence of *bZIP68* from wild-type genomic DNA was amplified as the native promoter to replace the 35S promoter in *1300221-Flag*. The CDS of *bZIP68* and its mutant forms were inserted between the *bZIP68* native promoter and the Flag tag. The recombinant vectors were introduced into rice via Agrobacterium (*EHA105*)-mediated transformation [41]. T3 homozygous plants identified by hygromycin selection and qPCR (Figure S1) were used in this study.

For osmotic stress analysis, rice seeds were sterilized with 5% sodium hypochlorite for 30 min and germinated in the dark at 28 °C in a constant-temperature incubator. Germinated seeds were transplanted into 96-well hydroponic boxes and grown in a growth chamber with 1/2 Hogland nutrient solution. Growth conditions were: 14 h light/10 h dark, 28 °C/24 °C, 60% humidity, with a light intensity of 200 μmol m^−2^ s^−1^ provided by white fluorescent tubes. 2-week-old seedlings were transferred to 1/2 Hogland nutrient solution with or without 20% PEG 6000 for 6 days. The relative malondialdehyde (MDA) content and the percentage of electrolyte leakage were determined from rice seedling leaves harvested four days after the related treatment [42].

### 4.2. Measurement of MDA Content

0.5 g of plant tissue was shredded and ground into a homogenate at low temperature with an appropriate amount of 10% trichloroacetic acid (TCA) and quartz sand. The homogenate was centrifuged at 4000× *g* for 20 min, and the supernatant was collected as the test sample solution. An equal volume of sample solution was mixed with 0.67% thiobarbituric acid (TBA) solution, and the mixture was incubated in a metal bath at 100 °C for 15 min for color development. The reaction was terminated immediately by ice bath, followed by another centrifugation. A blank control was set using an equal volume of ultrapure water instead of the sample solution for parallel treatment. Absorbance values were measured at three wavelengths (450 nm, 532 nm, 600 nm) by spectrophotometry. MDA concentration (μmol L^−1^) = 6.45 × (OD_532_ − OD_600_) − 0.56 × OD_450_; MDA content (μmol g^−1^) = V × C/W; where V is the volume of the extract, and W is the fresh weight of the sample.

### 4.3. Measurement of Electrolyte Leakage in Rice

Rice shoots were excised and immersed in deionized water for equilibration. After 2 h, the electrolyte conductivity of the equilibrated solution was measured as E_1_ by using a conductivity meter (DDS-11A, INESA, Shanghai, China). The samples were then boiled for 20 min, and the electrolyte conductivity was measured as E_2_ after cooling. The conductivity of deionized water was used as a control (E_3_) to calculate the relative electrolyte leakage of plant materials. Relative electrolyte leakage (%) = [(E_1_ − E_3_)/(E_2_ − E_3_)] ×100.

### 4.4. Expression and Purification of Recombinant Protein

The pET-28a vector was used for prokaryotic expression and purification. The *bZIP68-pET28a* construct was transformed into *BL21* competent cells (Vazyme, Nanjing, China). The cells were cultured with shaking at 37 °C and 200 rpm. When the OD600 of the bacterial culture reached 0.4–0.6, Isopropyl β-D-thiogalactoside (IPTG) was added to a final concentration of 0.1 mM, and protein expression was induced at 37 °C for 4 h. After centrifugation to collect the bacterial cells, the cell pellet was resuspended in PBS buffer and lysed by ultrasonication. The lysate was centrifuged at 12,000 rpm for 10 min, and the supernatant was collected for protein purification. The His-tagged bZIP68 recombinant protein was purified using a Ni-NTA prepacked column (Sangon Biotech, Shanghai, China), following the manufacturer’s instructions strictly.

### 4.5. Immunochemical Detection of Persulfidated Proteins

Persulfidation levels of proteins were detected using a modified Tag-switch method [18]. For in vitro assays, the purified recombinant protein was treated with NaHS, DTT, or K_2_S_x_ at the indicated concentrations, respectively. Methylsulfonyl benzothiazole (MSBT) was added to block non-target proteins, and persulfidated cysteines were then labeled with CN-biotin. Persulfidated proteins were detected by immunoblotting using an anti-biotin-HRP antibody (1:10,000; Abcam, Cambridge, UK). For in vivo assays, the *bZIP68-Flag* fusion protein was expressed in tobacco leaves via Agrobacterium-mediated infiltration. After treatment with K_2_S_x_ or H_2_O_2_, tobacco leaves were ground in liquid nitrogen. Total proteins were extracted using a buffer (25 mM Tris, 100 mM NaCl, 0.2% (*v*/*v*) Triton X-100, pH 8.0) supplemented with a complete protease inhibitor cocktail. A blocking buffer (50 mM MSBT dissolved in tetrahydrofuran) was added to the protein extract, and the mixture was incubated at 37 °C for 1 h to block free thiol groups. Proteins were precipitated with acetone, and the pellet was resuspended in a buffer (50 mM Tris, 2.5% (*w*/*v*) SDS, 20 mM CN-biotin, pH 8.0) and incubated at 37 °C for 3–4 h. After another round of acetone precipitation, the pellet was washed twice with 70% (*w*/*v*) acetone and resuspended in a buffer (50 mM Tris, 0.5% (*w*/*v*) SDS, pH 8.0).

Biotinylated proteins were purified by immunoprecipitation with 80 μL of streptavidin magnetic beads (Roche, Basel, Switzerland) at 25 °C for 1 h. After washing the beads three times, the bound proteins were eluted with an appropriate buffer, separated by 12% (*w*/*v*) SDS-PAGE, and transferred onto a polyvinylidene difluoride (PVDF) membrane. Target proteins were detected using an Anti-DYKDDDDK Tag Antibody (1:2000; HuaAn Biotech, Jinan, China).

### 4.6. Dual-LUC Assay

The *35S:bZIP68-GFP* construct and its point-mutated recombinant vectors were used as effector vectors. The recombinant vectors containing *COR413-TM1* and *pGreenII 0800-LUC* were used as reporter vectors, and the empty *35S:GFP* vector was used as the negative control. The recombinant plasmids were extracted and purified by large-scale plasmid preparation, and co-transformed into rice protoplasts. The protoplasts were incubated at 28 °C overnight in the dark for protein expression. For detection, proteins were extracted using the Dual Luciferase Reporter Assay Kit (Vazyme, DL101-01), and the luciferase activities were measured with a multimode microplate reader. The ratio of firefly luciferase activity to renilla luciferase activity was used to represent the relative expression level of the target gene regulated by the transcription factor.

### 4.7. Transcriptome Analysis

14-day-old rice seedlings were treated with or with 20% PEG 6000 for 12 h, and leaves were harvested for total RNA extraction. RNA-seq was performed at Gene Denovo (Guangzhou, China). The extracted mRNA is enriched using mRNA Capture Beads. After purification with beads, the mRNA is fragmented using high temperatures. The fragmented mRNA is then used as a template to synthesize the first strand of cDNA in a reverse transcription enzyme mixture system. While synthesizing the second strand of cDNA, end repair and A-tailing are completed. Next, adapters are ligated, and Hieff NGS^®^ DNA Selection Beads are used for purification to select target fragments. PCR library amplification is then performed, and finally, detection is carried out using the Illumina Novaseq X Plus. Hisat2 v2.0.5 was used to analyze the RNA-seq clean reads, referencing the rice genome (Ensembl_release58_IRGSP-1.0_Nipponbare). Genes with *p* < 0.05 and log2_ratio over 1 were identified as DEGs using DEseq2. GO analysis of these DEGs was conducted by using OmicShare online software (www.omicshare.com/tools/).

### 4.8. Real-Time Quantitative PCR (RT-qPCR)

The Total RNA was extracted using TRIzol reagent (Takara). RT-qPCR analysis was performed using the reverse-transcribed cDNA as the template and rice *OsACTIN1* as the internal control in a reaction mixture of 20 μL of SYBR^®^ Premix Ex Taq™ (TaKaRa Bio, Beijing, China) according to the manufacturer’s instructions. The relative gene expression levels were quantified using the 2^−ΔΔCt^ method based on three biological replicates. The primers used for specific genes are listed in Table S7.

### 4.9. Statistical Analysis

All data were analyzed using SPSS 23.0 (SPSS, Chicago, IL, USA). Comparisons were performed by independent sample *t*-test (two-tailed) or one-way ANOVA based on Duncan’s multiple range test (two-tailed).

## Data Availability

The original contributions presented in this study are included in the article/Supplementary Material. Further inquiries can be directed to the corresponding authors.

## Supplementary Materials

The following supporting information can be downloaded at: https://www.mdpi.com/article/10.3390/ijms27093841/s1.

## Abbreviations

The following abbreviations are used in this manuscript:

ROS	Reactive oxygen species
H_2_S	Hydrogen sulfide
MDA	Malondialdehyde
TCA	Trichloroacetic acid
TBA	Thiobarbituric acid
IPTG	Isopropyl β-D-thiogalactoside
DTT	Dithiothreitol
MSBT	Methylsulfonyl benzothiazole
PVDF	Polyvinylidene difluoride
