# Supplementary material for: Hydrogen Sulfide Primes bZIP68 via Persulfidation to Enhance Redox-Dependent Transcription and Adaptation to Osmotic Stress in Rice
Source: Int J Mol Sci. 2026 Apr 26;27(9):3841. doi: 10.3390/ijms27093841 (PMC13164137; doi:10.3390/ijms27093841)
Supplement: Supplementary file 1 [file ijms-27-03841-s001.zip › Supplemental Figure.pdf]

Supporting information

Hydrogen sulfide primes bZIP68 via persulfidation to enhance redox-dependent transcription and osmotic stress tolerance in rice

Figure S1.

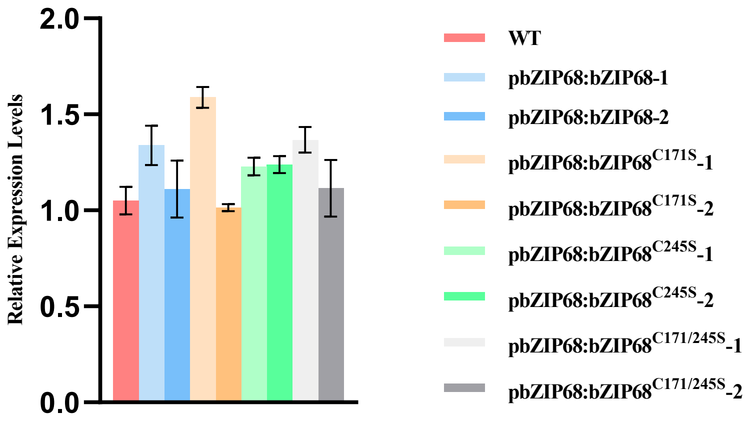

Figure S1. Characterization of complementary lines of *bZIP68*. The bar graph presents the relative expression levels of *bZIP68* (quantified via RT-qPCR, with rice *OsACTIN1* as the internal control) in WT and various *bZIP68* complementary lines: pbZIP68/bZIP68 (lines 1–2), pbZIP68/bZIP68^{C171S} (lines 1–2), pbZIP68/bZIP68^{C245S} (lines 1–2), and pbZIP68/bZIP68$^{C171S/C245S}$ (lines 1–2). Expression levels were normalized to that of WT. Error bars represent the standard deviation from three independent biological replicates.
